# The effects of hot‐water immersion on cardiovascular and cardiorespiratory health of healthy adults: A systematic review and meta‐analysis

**DOI:** 10.14814/phy2.70668

**Published:** 2026-01-28

**Authors:** Bruna Bittencourt Sotomaior, Ian B. Stewart, Patrick Rodrigues, Raul Osiecki, Geoffrey M. Minett

**Affiliations:** ^1^ School of Exercise and Nutrition Sciences Queensland University of Technology Brisbane Australia; ^2^ School of Sport and Human Movement University of Waikato Hamilton New Zealand; ^3^ Department of Physical Education Federal University of Parana Curitiba Brazil

**Keywords:** cardiorespiratory fitness, cardiovascular health, exercise mimetics, heat stress, hot water immersion, passive heating

## Abstract

Regular exercise benefits cardiovascular and cardiorespiratory health. Passive heating also induces cardiovascular responses, but its effect on cardiorespiratory fitness remains unclear. Given the overlapping physiological mechanisms between passive heating and exercise, hot‐water immersion may serve as an alternative strategy to prevent decline in cardiovascular function and fitness. This systematic review and meta‐analysis investigated the effects of hot‐water immersion (HWI) on cardiovascular health markers and cardiorespiratory fitness in healthy populations. A comprehensive search of six databases identified 20 studies. The meta‐analysis found that a single HWI session significantly increased heart rate (*N* = 10; mean difference [MD]: 28 bpm, 95% confidence interval [CI]: 19–36.2, *p* < 0.0001) and decreased diastolic (*N* = 6; MD: −5 mmHg, 95% CI: −9 to −1, *p* = 0.015) and mean arterial blood pressure (*N* = 4; MD: −7 mmHg, 95% CI: −12 to −1, *p* = 0.03). Repeated immersion reduced resting heart rate (*N* = 5; MD: −3 bpm, 95% CI: −6 to −1 *p* = 0.01). No significant effects were observed for other cardiovascular markers, and only one study reported data on cardiorespiratory fitness. Overall, our findings indicate inconclusive beneficial effects of HWI on cardiovascular health markers and further research is needed, especially on cardiorespiratory fitness.

## 1 INTRODUCTION

Cardiovascular disease (CVD) remains the leading cause of death worldwide. In 2022, an estimated 19.8 million people died from CVDs, accounting for approximately a third of global deaths (WHO, 2025). Beyond mortality, CVDs impose a considerable economic strain on healthcare systems, with costs estimated at US$863 billion in 2010, and projected to exceed US$1044 billion by 2030 (Rittiphairoj et al., 2025). Although many CVDs can be prevented by addressing risk factors such as physical inactivity, 31.3% of adults (Strain et al., 2024) do not meet the recommendations for physical activity (Bull et al., 2020). The reason for that is multifactorial, with individual, environmental, social, and cultural factors influencing physical activity levels (Bauman et al., 2012). Accordingly, alternative preventive strategies that offer comparable health benefits and achieve higher levels of adherence are needed, with passive heating emerging as a promising approach. Importantly, early vascular changes and declines in cardiorespiratory fitness can begin in young adulthood (Gabriel et al., 2022; Guo et al., 2025), highlighting the value of interventions that promote vascular health and cardiorespiratory fitness before marked deterioration occurs.

Passive heat therapies have traditionally been used for leisure and relaxation, but evidence suggests they may offer health benefits. More recently, it has been proposed that passive heating shares common physiological mechanisms with exercise (Cullen et al., 2020). The thermal stress induced by exercise acts as an endogenous stimulus for enhancing skeletal muscle function (Hawley et al., 2014; Philp et al., 2021), improving metabolic health (Archer et al., 2018), and promoting cardiovascular adaptations (Adams & Linke, 2019; Li et al., 2020). Similarly, passive heat exposure has been shown to induce mitochondrial adaptations (Marchant et al., 2023), increase extracellular heat shock protein (eHSP) expression (Faulkner et al., 2017), improve glucose metabolism (Pallubinsky et al., 2020), promote muscular hypertrophy (Rodrigues et al., 2020), and enhance muscle contractile function (Rodrigues et al., 2023). However, these outcomes are not consistently reported across the literature (Hesketh et al., 2019; Stadnyk et al., 2018).

Among these benefits, the cardiovascular effects of passive heating have received particular attention (Kohara et al., 2018; Laukkanen et al., 2015), as explored in previous reviews (Pizzey et al., 2021), particularly those focusing on sauna bathing (Hussain & Cohen, 2018; Källström et al., 2018; Laukkanen & Kunutsor, 2024). Most recently, Rodrigues, O'Connor, et al. (2025) conducted a systematic review of passive heat therapy in older adults with cardiovascular disease, reporting improvements in ejection fraction, flow‐mediated dilation, and disease severity markers such as brain natriuretic peptide and New York Heart Association classification. Importantly, their review aggregated multiple heat modalities, including Waon therapy, balneotherapy, and sauna use, without isolating the effects of hot‐water immersion (HWI), despite its physiological mechanisms and practical advantages.

Unlike air‐based heating, HWI uses water's superior thermal conductivity to increase core temperature more efficiently and comfortably, often at lower temperatures (Atencio et al., 2025). This may enhance tolerability and adherence, as evidenced by studies where participants completed HWI protocols more consistently than sauna sessions (Campbell et al., 2022). However, tolerability is influenced by several factors, such as immersion duration, water temperature, body surface area submerged, and participant characteristics. Additionally, HWI exerts hydrostatic pressure on the body, which alters cardiovascular dynamics by increasing cardiac output and mean arterial pressure while reducing peripheral vascular resistance (Carter et al., 2014; Simmons et al., 2017). While maintaining high water temperatures may incur energy costs, HWI remains relatively accessible compared to other structured passive heating modalities such as sauna bathing or climatic chamber exposure, which often require specialized equipment or facilities. HWI can be performed using basic household infrastructure (e.g., a bathtub), making it a feasible option for home‐based use. Moreover, its affordability also makes HWI a relevant option for populations at elevated cardiovascular risk, particularly those with lower socioeconomic status in high‐income countries (Clark et al., 2009).

Acute passive heat exposure elevates core and skin temperatures, eliciting thermoregulatory vasodilation and redistribution of blood flow toward the skin to enhance heat dissipation (Gravel et al., 2021; Rowell et al., 1970). To accommodate the increased peripheral blood flow, cardiac output rises, primarily mediated by an elevation in heart rate through autonomic modulation, as stroke volume generally remains unchanged (Crandall & Wilson, 2015). Increased myocardial contractility helps maintain blood pressure despite a reduction in total peripheral resistance, ultimately leading to decreased cardiac filling pressures (Crandall & Wilson, 2015). Repeated over time, these acute responses may underpin long‐term cardiovascular adaptations. Indeed, epidemiological evidence supports this notion, with frequent sauna use associated with reduced risk of fatal cardiovascular and all‐cause mortality events (Laukkanen et al., 2015), and habitual hot spring bathing inversely linked with coronary heart disease, cerebrovascular disease, hypertension, and diabetes (Ukai et al., 2020). These associations may be explained by the improved vascular properties that follow repeated heat exposure. Endothelial function, a key determinant of vascular health and CVD risk, is commonly assessed via flow‐mediated dilation and arterial stiffness. Brunt, Howard, et al. (2016) demonstrated that 8 weeks of repeated hot‐water immersion at 40.5°C improved mean arterial blood pressure, brachial artery flow‐mediated dilation, and central arterial stiffness to a degree similar to the improvements typically reported in exercise‐training studies involving sedentary individuals.

While these findings suggest that hot‐water immersion may elicit exercise‐like cardiovascular adaptations, the specific exercise dose it reflects remains unclear, as studies have used varied comparators. For instance, Thomas et al. (2016) matched hot‐water immersion duration (i.e., 30 min) to treadmill running at 65%–75% of the age‐predicted maximum heart rate. Alternatively, Amin et al. (2021) compared 30 min of hot‐water immersion with graded semi‐recumbent stepping exercise, where the exercise workload was matched to the increase in cardiac output and heart rate observed at the end of the heating period. Regular exercise is known to enhance cardiorespiratory fitness, which serves as a protective factor against CVDs. Bailey et al. (2016) reported a ~2.3 mL.kg^−1^.min^−1^ increase in VO_2peak_ in healthy females following both aerobic exercise and whole‐body water immersion over 8 weeks, evidencing that passive heating may also improve fitness. Importantly, 30 min of hot‐water immersion at 40°C, either following exercise or another HWI bout, is perceived as more enjoyable than passive rest after exercise, suggesting that the enjoyment and tolerability of HWI may promote adherence (Steward et al., 2024), particularly in populations with limited exercise capacity (Akerman et al., 2019).

Despite these promising findings, previous systematic reviews have not specifically examined the effects of hot‐water immersion on cardiovascular function as well as cardiorespiratory fitness, particularly in healthy populations. Hot‐water immersion (HWI) is a widely accessible, cost‐effective, and easily implemented modality that may be effective as a preventive strategy to mitigate early declines in cardiovascular function and cardiorespiratory fitness before significant deterioration occurs. This is especially relevant given that subtle vascular impairments and reductions in CRF can emerge in young, otherwise healthy adults, and may contribute to long‐term cardiovascular risk if left unaddressed. Therefore, this systematic review and meta‐analysis aimed to address this gap by examining the effects of a single and repeated hot‐water immersion exposures on markers of cardiovascular function and cardiorespiratory fitness in healthy adults, allowing for a clearer understanding of HWI's specific benefits and limitations and better guidance for real‐world application.

## 2 METHODS

This systematic review and meta‐analysis performed according to the Preferred Reporting Items for Systematic Review and Meta‐Analysis (PRISMA, Supplementary File S1) (Page et al., 2021). The protocol was registered on PROSPERO with the identification number CRD42023412125. The subgroup analysis, as prespecified in the protocol, was not undertaken because the available data were insufficient. No amendments were made to the registered protocol.

### 2.1 Data sources and search strategy

Six electronic databases (PubMed, Web of Science, Scopus, EMBASE, CINAHL, and SPORTDiscus) were searched for relevant literature up to 4 May 2025. Search terms or keywords related to passive heating (“immersion,” “water,” “bath,” “hot temperature,” “hyperthermia,” “hot water immersion,” “passive heat,” “heat stress,” “heat therapy,” “heat acclimation,” “heat exposure,”) cardiovascular health (“blood pressure,” “cardiac output,” “stroke volume,” “heart rate,” “plasma volume,” “blood plasma volume,” “blood flow velocity,” “arterial stiffness,” “cardiovascular,” “endothelial function,” “shear rate,” “artery flow mediated dilation,” “artery diameter,”) and cardiorespiratory health (“cardiorespiratory fitness,” “oxygen consumption,” “maximal oxygen consumption,” “vo2,” “vo2max,” “aerobic fitness”) were combined, and search terms were adjusted according to the databases' specifications. The full search strategy is found in Appendix S2. No language and publication date restrictions were applied. In addition, reference lists of included studies were screened, and citation tracking was conducted using Google Scholar to identify any relevant articles not captured in the initial database search.

Inclusion criteria included: (1) randomized, non‐randomized, and cross‐over studies; (2) adults ≥18 years of age; (3) healthy population; (3) immersion in hot water (e.g., part‐ or whole‐body); (4) control group or control condition. Studies were excluded based on the following criteria: (1) review papers and abstracts; (2) humans <18 years of age; (3) pregnant women; (4) diagnosis of CVD and other diseases; (5) other passive heating interventions rather than hot‐water immersion; (6) use of hot‐water immersion as a recovery method for sport or exercise; (7) animal, tissue, or cell samples. No restrictions were placed on sex, level of physical activity, and duration of intervention (e.g., single or repeated exposures).

### 2.2 Study selection

After duplicates were removed using referencing software (EndNote 20, Clarivate Analytics, Philadelphia, USA), two reviewers (BBS and GM) independently and blindly screened titles and abstracts based on the inclusion and exclusion criteria using the web tool Rayyan (Ouzzani et al., 2016). The same investigators screened the full text for eligibility. Any disagreements were resolved through discussion with a third reviewer (PR).

### 2.3 Data extraction

Data were extracted by one reviewer (BBS) and checked for accuracy by another reviewer (GM). Data were extracted in an Excel spreadsheet and included: study characteristics (title, first author name, year of publication, journal, country, and study design); sample characteristics (number of participants, age, sex, height, body mass, body mass index, population [healthy or clinical]); intervention details (water temperature, session duration [min], frequency [days], length [weeks], total sessions, and depth of immersion); cardiovascular outcomes (cardiorespiratory fitness [i.e., direct or indirect assessment of maximal oxygen consumption or peak oxygen consumption], physical performance [e.g., functional capacity tests, performance tasks and tolerance times], blood pressure, heart rate, cardiac output, stroke volume, plasma volume, endothelial function [assessed by artery flow‐mediated dilatation], shear rate [assessed by artery diameter and blood flow], or arterial stiffness [assessed by pulse wave velocity]). In case of incomplete or unreported data, the primary author was contacted by e‐mail. When results were reported only in figures, PlotDigitizer (plotdigitizer.com) was used to retrieve the data.

### 2.4 Risk of bias assessment

Two reviewers (PR and IS) independently assessed the risk of bias in the included studies following the Cochrane guidelines. Any disagreement was resolved by a third reviewer (BBS). The Revised Cochrane risk‐of‐bias tool (RoB 2) was used to assess randomized studies. This tool evaluates six possible sources of bias: (1) bias arising from the randomization process; (2) bias arising from period and carryover effects; (3) bias due to deviations from the intended interventions; (4) bias due to missing outcome data; (5) bias in the measurement of the outcome; (6) bias in the selection of the reported result, proposing a judgment of “low,” “some concerns,” or “high” risk of bias. The Risk of Bias in Non‐randomized Studies of Interventions tool (ROBINS‐I) was used for non‐randomized studies. Seven domains are assessed: (1) bias due to confounding; (2) bias in the selection of participants into the study; (3) bias in classification of intervention; (4) bias due to deviations from intended interventions; (5) bias due to missing data; (6) bias in the measurement of outcomes; and (7) bias in selection of the reported result, and classified as “low risk,” “moderate risk,” “serious risk,” “critical risk,” or “no information.” The risk of bias plots were created in the Shiny web app (McGuinness & Higgins, 2021).

### 2.5 Data analysis

The mean value, standard deviation (SD) and number of participants (*N*) were extracted. In studies reporting the standard error (SE), the SD was obtained by multiplying the SE of the mean by the square root of the sample size ($\mathrm{SD}=\mathrm{SE}\times \sqrt{n}$). Mean differences and 95% confidence intervals (CI) were calculated for each outcome measurement. In studies that reported subgroups (i.e., more than one intervention group or control group), the effect sizes were combined and analyzed as a single intervention. Specifically, the effect sizes for heart rate, blood pressure and cardiac output for the thermoneutral immersion and time control groups from (Cui et al., 2022) were combined. The mean and SD were calculated according to the Cochrane Handbook for Systematic Reviews of Interventions (Chapter 6).

Considerable between‐study heterogeneity was anticipated, for example, due to differences in study design, so a random‐effects model was used to pool effect size estimates. The restricted maximum likelihood estimator was used to estimate between‐study variance, *τ* ^2^. The Knapp–Hartung method was used to construct confidence intervals around the pooled effect size. Estimates from the studies were inverse variance weighted. For studies that did not report a variable of interest, the missing data were not included in the model to pool the effect sizes.

We report relative (*I* ^2^) and absolute (*τ* ^2^) heterogeneity. A prediction interval was also computed to estimate the likely interval range of effect sizes that future studies of hot‐water immersion could expect when applied in similar populations (Borg et al., 2024). Prediction intervals were calculated based on a *t*‐distribution with *k*–2 degrees of freedom, where *k* is the number of studies in the meta‐analysis (Borg et al., 2024). A sensitivity analysis was performed using the leave‐one (effect size estimate)‐out method to explore each study's influence on the pooled effect size. Using this approach, the meta‐analysis results were calculated *m* times, each time leaving out one effect size and recalculating the overall mean difference and between‐study heterogeneity. Counter‐enhanced funnel plots were visually inspected to examine small‐sample bias (Peters et al., 2008). Egger's regression test was also used to investigate small sample bias (Egger et al., 1997). All analyses were performed in R (v 4.3.1; R Core Team, 2023) using meta (Balduzzi et al., 2019), metafor (Viechtbauer, 2010), and dmetar (Harrer et al., 2019) packages.

## 3 RESULTS

**FIGURE 1:**
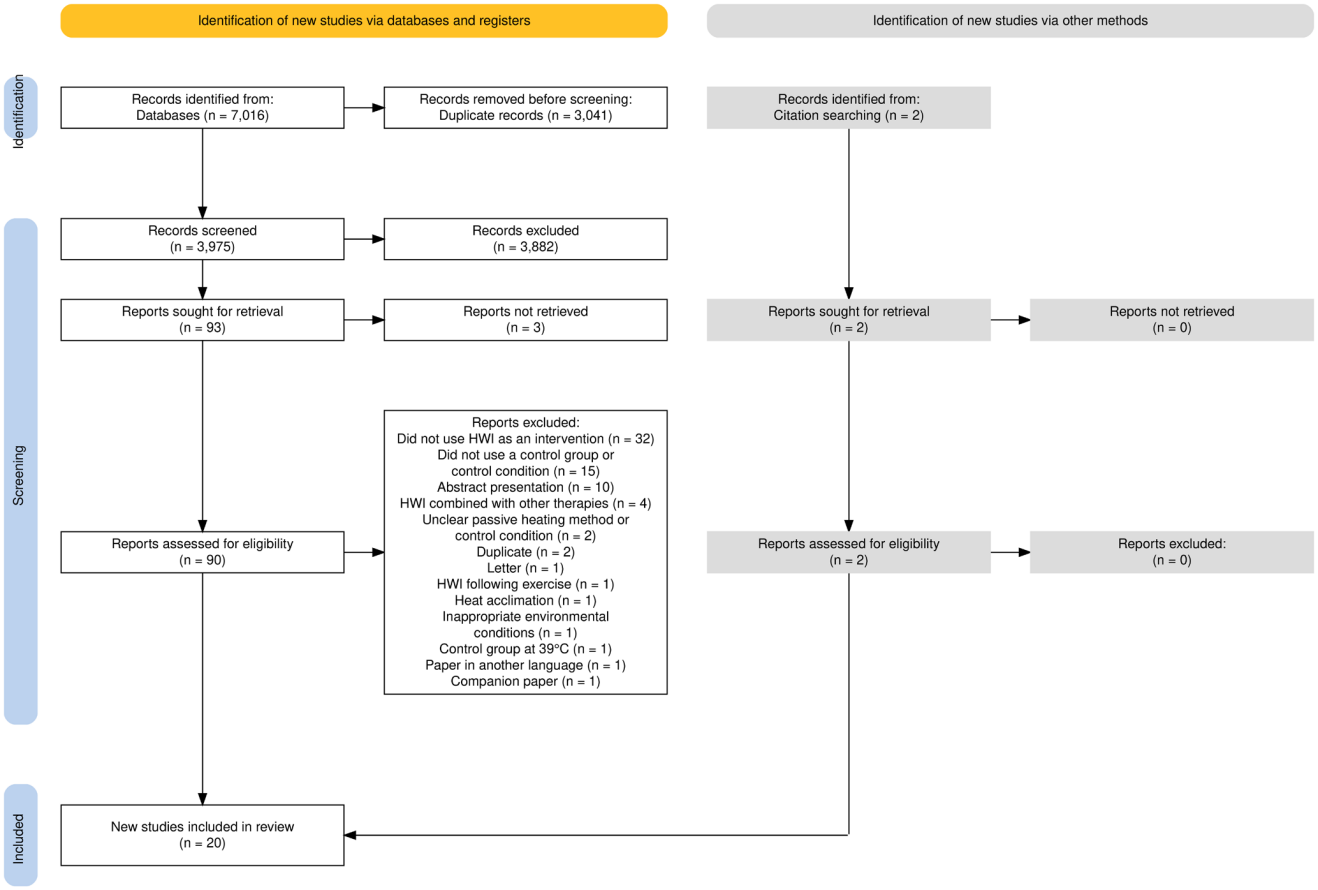
Study flow diagram.

The search returned 7016 references before duplicates were removed (*n* = 3041). Titles and abstracts of the remaining 3975 articles were screened based on the inclusion/exclusion criteria. Ninety‐three studies were considered eligible for full‐text reading, and 20 original articles were included in this review (15 were included in the meta‐analysis) (Figure 1).

### 3.1 Participant characteristics

A total of 384 participants (male, *n* = 210; female, *n* = 151; sex not specified, *n* = 23) were included. Ten studies included both sexes (Brazaitis & Skurvydas, 2010; Brunt, Eymann, et al., 2016; Brunt, Howard, et al., 2016; Brunt, Jeckell, et al., 2016; Campbell et al., 2022; Cheng et al., 2021, 2025; Cui et al., 2022; Engelland et al., 2020; Su et al., 2024), seven studies included only males (Bellini et al., 2025; Eimantas et al., 2022; Kingma et al., 2021; Maley et al., 2023; Mansfield et al., 2021; Miwa et al., 1994; Treigyte et al., 2024), and two studies included only female participants (Hu et al., 2012; Kudo et al., 2019). Baranauskiene et al. (2023) did not report the number of males and females, and did not respond to correspondence seeking clarification.

All studies examined healthy populations. Seventeen studies included young adults (Bellini et al., 2025; Brazaitis & Skurvydas, 2010; Brunt, Eymann, et al., 2016; Brunt, Howard, et al., 2016; Brunt, Jeckell, et al., 2016; Campbell et al., 2022; Cheng et al., 2021, 2025; Eimantas et al., 2022; Engelland et al., 2020; Kingma et al., 2021; Kudo et al., 2019; Maley et al., 2023; Mansfield et al., 2021; Miwa et al., 1994; Su et al., 2024; Treigyte et al., 2024), one included only older adults (>57 years old; Cui et al., 2022), and two studies included both (Baranauskiene et al., 2023; Hu et al., 2012). To distinguish the data between young and older adults, references to Hu et al. (2012) will be cited as Hu et al. (1) for young adults and Hu et al. (2) for older adults. Participants were described as physically or recreationally active in 12 studies (Baranauskiene et al., 2023; Bellini et al., 2025; Brazaitis & Skurvydas, 2010; Brunt, Jeckell, et al., 2016; Campbell et al., 2022; Cheng et al., 2021, 2025; Eimantas et al., 2022; Maley et al., 2023; Mansfield et al., 2021; Su et al., 2024; Treigyte et al., 2024), sedentary in three studies (Brunt, Eymann, et al., 2016; Brunt, Howard, et al., 2016; Hu et al., 2012), and five studies did not report the physical fitness levels of the participants (Cui et al., 2022; Engelland et al., 2020; Kingma et al., 2021; Kudo et al., 2019; Miwa et al., 1994).

### 3.2 Intervention characteristics

The intervention details are displayed in Table 1. Ten studies applied whole‐body water immersion, here defined as immersion to the neck, shoulders or chest (Bellini et al., 2025; Brunt, Eymann, et al., 2016; Brunt, Howard, et al., 2016; Brunt, Jeckell, et al., 2016; Campbell et al., 2022; Cui et al., 2022; Eimantas et al., 2022; Kingma et al., 2021; Maley et al., 2023; Su et al., 2024), nine studies employed lower‐body immersion, defined as immersion at or below the waist line, including knee‐ or foot‐only immersion (Baranauskiene et al., 2023; Brazaitis & Skurvydas, 2010; Cheng et al., 2021; Cheng et al., 2025; Engelland et al., 2020; Hu et al., 2012; Mansfield et al., 2021; Miwa et al., 1994; Treigyte et al., 2024) and one study applied hand immersion (Kudo et al., 2019). The temperature of the interventions ranged from 37.5°C to 45°C. A time control group only was applied in nine studies (Baranauskiene et al., 2023; Brazaitis & Skurvydas, 2010; Brunt, Jeckell, et al., 2016; Cheng et al., 2021, 2025; Hu et al., 2012; Kudo et al., 2019; Su et al., 2024; Treigyte et al., 2024), a thermoneutral water immersion only in nine studies (Bellini et al., 2025; Brunt, Eymann, et al., 2016; Brunt, Howard, et al., 2016; Campbell et al., 2022; Engelland et al., 2020; Kingma et al., 2021; Maley et al., 2023; Mansfield et al., 2021; Miwa et al., 1994), and two studies employed both control groups (Cui et al., 2022; Eimantas et al., 2022). The temperature for the thermoneutral water immersion ranged from 33°C to 36.5°C, and the immersion depth was matched between conditions in all studies.

**TABLE 1:** Summary of the hot‐water immersion and control condition intervention details of the studies included in the systematic review and meta‐analysis.

Authors (years)	Groups	Intervention details					
		Type	Temperature	Duration (min)	Frequency (days)	Length (weeks)	Total sessions
Baranauskiene et al. (2023)	HWI	Immersion up to the waist level	43°C	69.14 ± 6.20	1	‐	1
	Control	Time control	‐	89.50 ± 4.90	1	‐	1
Bellini et al. (2025)	HWI	Immersion to the clavicle line, then immersion to the umbilicus for 30 min	39.1 ± 0.3°C	60	1	‐	1
	Control		36 ± 0.3°C	60	1	‐	1
Brazaitis and Skurvydas (2010)	HWI	Immersion up to the waist level	44°C	45	‐	2	7
	Control	Time control	‐	‐	‐	‐	‐
Brunt, Eymann, et al. (2016)	HWI	Immersion up to the shoulder for ~25–30 min, then immersion to waist level for 60 min	40.5°C	90	4–5	8	36
	Control		36°C	90	4–5	8	36
Brunt, Howard, et al. (2016)	HWI	Immersion up to the shoulder for ~25–30 min, then immersion to waist level for 60 min	40.5°C	90	4–5	8	36
	Control		36°C	90	4–5	8	36
Brunt, Jeckell, et al. (2016)	HWI	Immersion up to the shoulder for ~25–30 min, then immersion to waist level for 60 min	40.5°C	60	1	‐	1
	Control	Time control	‐	60	1	‐	1
Campbell et al. (2022)	HWI	Days 1 and 5: immersion to nipple height for 30 min, followed by shallower immersion to maintain *T*_re_	40.1 ± 0.2°C	60	5	‐	5
		Days 2–4: immersion to the neck until *T*_re_ = +1.5°C followed by shallower immersion to maintain *T*_re_					
	Control		36.5 ± 0.2°C	60	5	‐	5
Cheng et al. (2021)	HWI	Immersion up to the ankles in a supine, bent‐leg position	45°C	45	1	‐	1
	HWI	Immersion up to the knees in a supine, bent‐leg position	45°C	45	1	‐	1
	Control	Time control	‐	45	1	‐	1
Cheng et al. (2025)	HWI	Lower‐limb (footbath)	42.8°C	45	3	8	24
	Control	Time control		‐	‐	‐	‐
Cui et al. (2022)	HWI	Immersion up to the mid‐chest	41°C	30	5	4	20
	Control		34°C–35°C	30	5	4	20
Eimantas et al. (2022)	HWI	Whole‐body immersion	45°C	5	‐	‐	1
	Control	Whole‐body immersion	37°C	5	‐	‐	1
	Control	Time control	‐	5	‐	‐	1
Engelland et al. (2020)	HWI	Lower legs (~33 cm) immersion	42°C	60	‐	‐	1
	Control		33°C	60	‐	‐	1
Hu et al. (2012)	HWI	Immersion up to the knees	41°C–43°C	30	‐	‐	1
	Control	Time control	‐	30	‐	‐	1
Kingma et al. (2021)	HWI	Head‐out water immersion	38°C	60	‐	‐	1
	Control		36°C	60	‐	‐	1
Kudo et al. (2019)	HWI	Hand immersion	40°C	10	‐	‐	1
	Control	Time control	‐	‐	‐	‐	1
Maley et al. (2023)	HWI	Immersion to the clavicle for 30 min, then immersion to the umbilicus	40.5 ± 0.5°C	120	‐	‐	1
	Control		35.9 ± 0.6°C	120	‐	‐	1
Mansfield et al. (2021)	HWI	Immersion up to the waist level	42°C	60	‐	‐	1
	Control		36°C	60	‐	‐	1
Miwa et al. (1994)	HWI	Squatting position	40°C	60	‐	‐	1
	Control		34.5°C	60	‐	‐	1
Su et al. (2024)	HWI	Immersion up to the neck	37.5°C	60	‐	‐	1
	Control	Time control	‐	60	‐	‐	1
Treigyte et al. (2024)	HWI	Immersion up to the waist level	44°C–45°C	45	‐	‐	1
	Control	Time control	‐	45	‐	‐	1

The immersion duration ranged from 5 min (Eimantas et al., 2022), 10 min (Kudo et al., 2019), 30 min (Cui et al., 2022; Hu et al., 2012), 45 min (Brazaitis & Skurvydas, 2010; Cheng et al., 2021, 2025; Treigyte et al., 2024), 60 min (Bellini et al., 2025; Brunt, Jeckell, et al., 2016; Campbell et al., 2022; Engelland et al., 2020; Kingma et al., 2021; Mansfield et al., 2021; Miwa et al., 1994; Su et al., 2024), 90 min (Brunt, Eymann, et al., 2016; Brunt, Howard, et al., 2016), to 120 min (Maley et al., 2023). In one study, the duration was based on the time for the rectal temperature to reach 39.5°C for the young group and 39°C for the older group (Baranauskiene et al., 2023). Regarding the length of intervention, 14 studies administered a single exposure (Baranauskiene et al., 2023; Bellini et al., 2025; Brunt, Jeckell, et al., 2016; Cheng et al., 2021; Eimantas et al., 2022; Engelland et al., 2020; Hu et al., 2012; Kingma et al., 2021; Kudo et al., 2019; Maley et al., 2023; Mansfield et al., 2021; Miwa et al., 1994; Su et al., 2024; Treigyte et al., 2024), and six studies analyzed repeated exposure to hot‐water immersion (Brazaitis & Skurvydas, 2010; Brunt, Eymann, et al., 2016; Brunt, Howard, et al., 2016; Campbell et al., 2022; Cheng et al., 2025; Cui et al., 2022). In the repeated exposure studies, the total number of sessions varied between five (Campbell et al., 2022), seven (Brazaitis & Skurvydas, 2010), 20 (Cui et al., 2022), 24 (Cheng et al., 2025), and 36 sessions (Brunt, Eymann, et al., 2016; Brunt, Howard, et al., 2016).

### 3.3 Risk of bias

**FIGURE 2:**
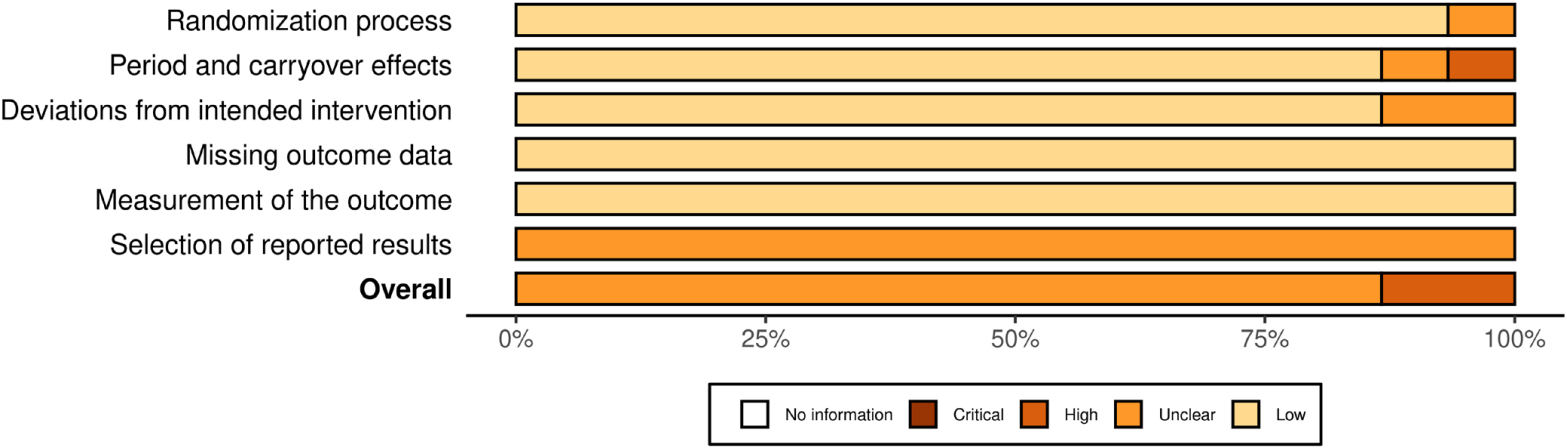
General risk of bias analysis in randomized studies presented as percentages across all included studies.

**FIGURE 3:**
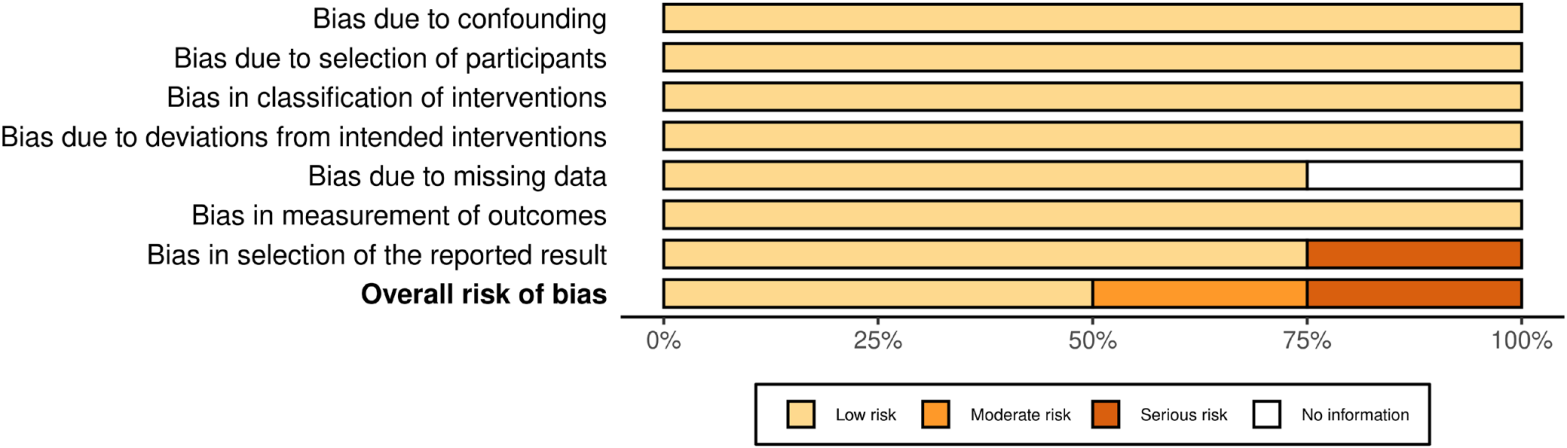
General risk of bias analysis in non‐randomized studies presented as percentages across all included studies.

**FIGURE 4:**
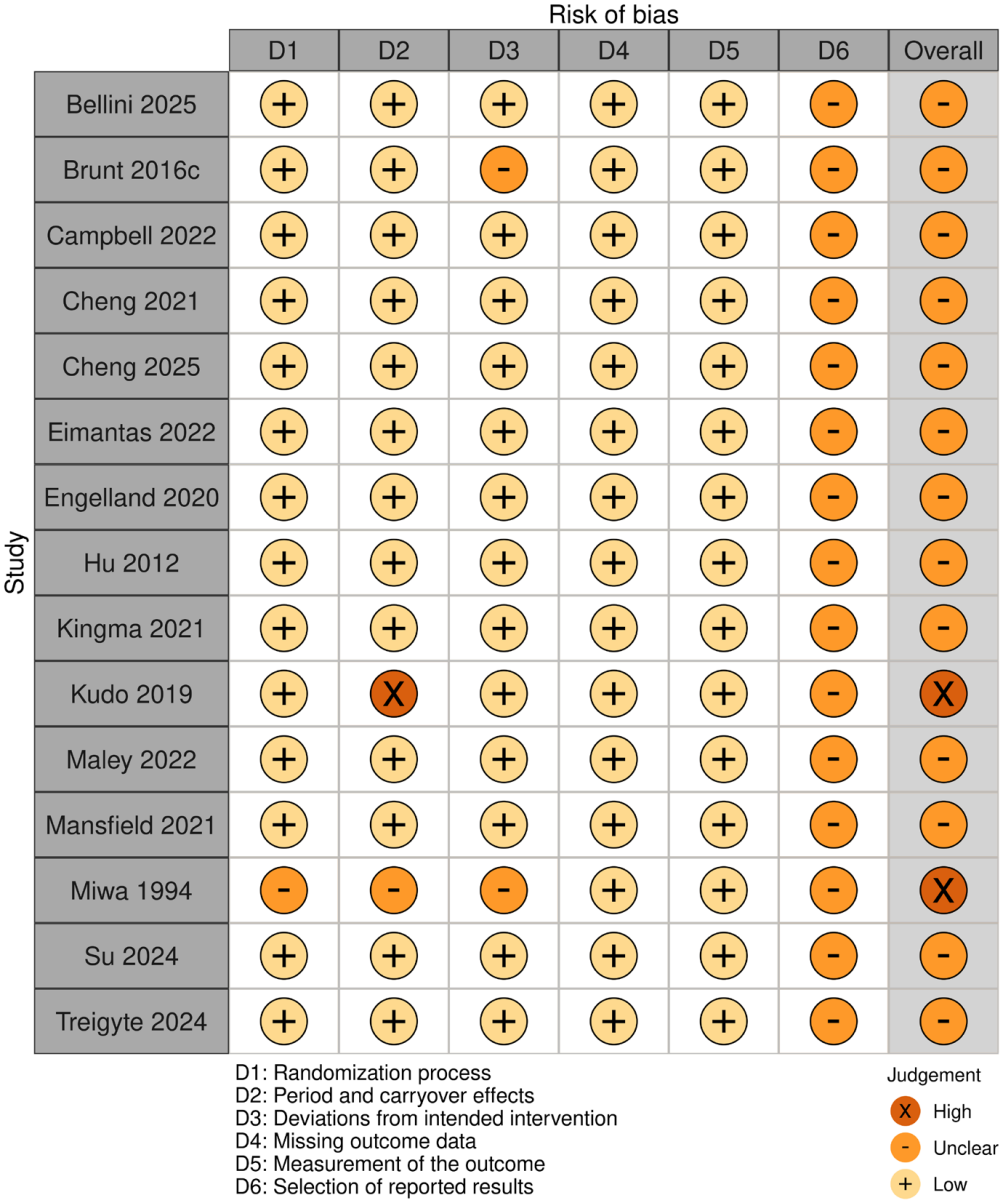
Detailed risk of bias analysis in each domain for each randomized study.

**FIGURE 5:**
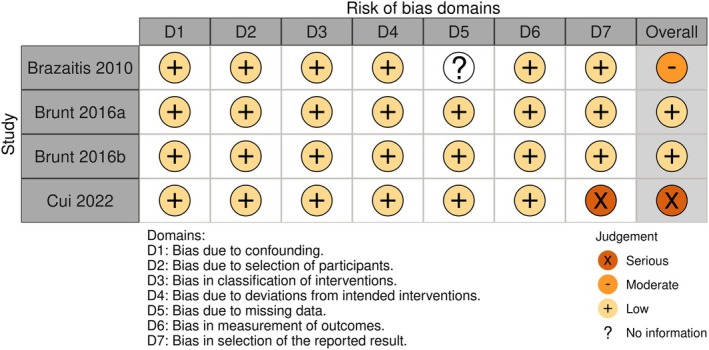
Detailed risk of bias analysis in each domain for each non‐randomized study.

The overall risk of bias analysis is shown in Figures 2 and 3. Sixteen studies were analyzed using the risk‐of‐bias tool (RoB 2) for randomized trials (Baranauskiene et al., 2023; Bellini et al., 2025; Brunt, Jeckell, et al., 2016; Campbell et al., 2022; Cheng et al., 2021, 2025; Eimantas et al., 2022; Engelland et al., 2020; Hu et al., 2012; Kingma et al., 2021; Kudo et al., 2019; Maley et al., 2023; Mansfield et al., 2021; Miwa et al., 1994; Su et al., 2024; Treigyte et al., 2024) (Figure 2), and in four studies, the risk of bias in non‐randomized studies–of interventions (ROBINS‐I) assessment tool was employed (Brazaitis & Skurvydas, 2010; Brunt, Eymann, et al., 2016; Brunt, Howard, et al., 2016; Cui et al., 2022) (Figure 3). A detailed risk of bias analysis in each domain for each study is shown in Figures 4 and 5. Most studies (15 of 20) were classified as having some concerns or a moderate risk of bias due to a lack of information in selecting reported results. Three studies were scored as having a high or serious risk of bias (Cui et al., 2022; Kudo et al., 2019; Miwa et al., 1994), and two studies were classified as having a low risk of bias (Brunt, Eymann, et al., 2016; Brunt, Howard, et al., 2016).

### 3.4 Heart rate

All 20 studies reported heart rate outcomes. Twelve studies measured heart rate using a chest strap (Baranauskiene et al., 2023; Brazaitis & Skurvydas, 2010; Brunt, Eymann, et al., 2016; Brunt, Howard, et al., 2016; Brunt, Jeckell, et al., 2016; Campbell et al., 2022; Eimantas et al., 2022; Kingma et al., 2021; Maley et al., 2023; Mansfield et al., 2021; Su et al., 2024; Treigyte et al., 2024), four studies used an electrocardiogram (Bellini et al., 2025; Cheng et al., 2021; Engelland et al., 2020; Miwa et al., 1994), two studies used an electric blood pressure monitor (Cheng et al., 2025; Hu et al., 2012), one study used a transthoracic echocardiography/Doppler (Cui et al., 2022), and one study used a heart rhythm scanner (Kudo et al., 2019).

Due to missing data, five studies were excluded from the meta‐analysis. One study did not measure heart rate in the control condition (Brunt, Jeckell, et al., 2016), and in three studies, the authors did not reply to our request (Baranauskiene et al., 2023; Brazaitis & Skurvydas, 2010; Eimantas et al., 2022). Kudo et al. (2019) were excluded from the meta‐analysis due to substantial heterogeneity, which violates meta‐analytic assumptions and risks introducing bias into pooled effect estimates. Ten studies assessed heart rate at the end of the single immersion exposure (Bellini et al., 2025; Cheng et al., 2021; Engelland et al., 2020; Hu et al., 2012; Kingma et al., 2021; Maley et al., 2023; Mansfield et al., 2021; Miwa et al., 1994; Su et al., 2024; Treigyte et al., 2024), and five studies measured resting heart rate following the repeated hot‐water immersion intervention (Brunt, Eymann, et al., 2016; Brunt, Howard, et al., 2016; Campbell et al., 2022; Cheng et al., 2025; Cui et al., 2022).

**FIGURE 6:**
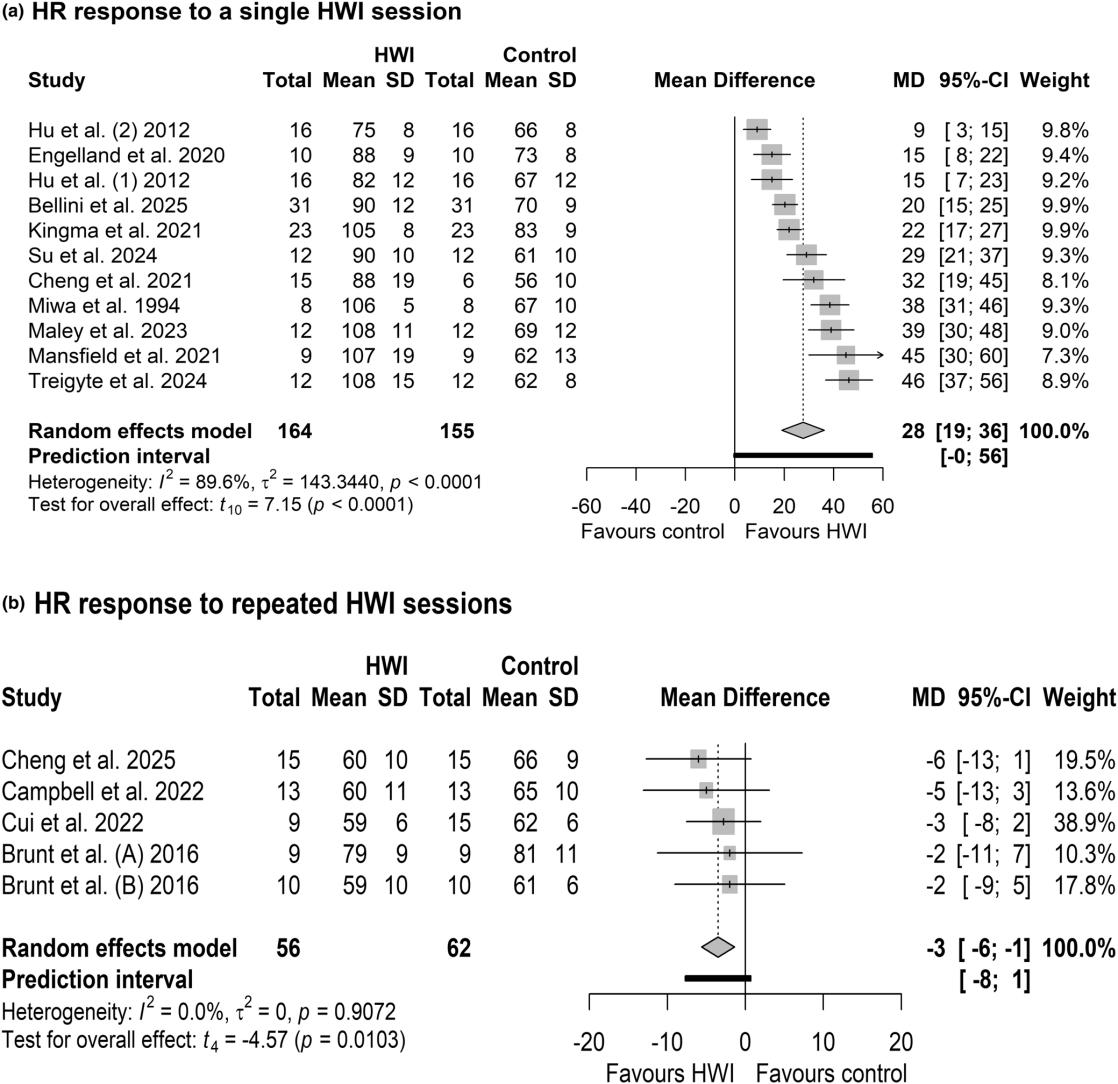
Forest plot of comparison: Hot‐water immersion (HWI) versus control (no HWI or thermoneutral water immersion [TWI]), outcome: (a) Heart rate response at the end of a single HWI session; (b) Resting heart rate response after repeated HWI intervention–random effects analysis.

Evidence shows that heart rate increased at the end of the single hot‐water immersion compared with control conditions (mean difference [95% confidence interval]: 28 bpm [19, 36.2], *p* < 0.0001; Figure 6a). However, the analysis showed significant heterogeneity (*I* ^2^ = 89.6%, *τ* ^2^ = 141, *p* < 0.0001), and the prediction interval (−0 to 56 bpm) indicates that future studies may expect large effects. Long‐term exposure to hot water reduced resting heart rate compared to control conditions (−3 bpm [−6, −1], *p* = 0.01; Figure 6b). Despite low heterogeneity (*I* ^2^ = 0%, *τ* ^2^ = 0, *p* = 0.09), a wide prediction interval was observed (PI: −8, 1).

A leave‐one‐out analysis was conducted to assess the sensitivity of the meta‐analytic estimates to each study (Table S1). For the single interventions, the mean differences ranged from 26 to 29 across the iterations, with a relatively consistent effect size observed regardless of which study was removed. The 95% CI for the mean difference remained consistently wide, indicating uncertainty regarding the magnitude of the effect. The prediction intervals were also wide across all iterations and often included positive and negative values. However, when Hu et al. (2) were excluded, the prediction interval ranged from 5 to 54. These wide prediction intervals suggest that, while the average effect size is relatively consistent, future observations may vary widely. The mean difference for the repeated interventions ranged from −3 to −4 across iterations, with a consistent effect size and prediction intervals, and often including positive and negative values.

### 3.5 Blood pressure

Eleven studies reported the effect of hot‐water immersion on blood pressure. Systolic and diastolic blood pressure (SBP, DBP) were acutely assessed in six studies (Bellini et al., 2025; Cheng et al., 2021; Hu et al., 2012; Maley et al., 2023; Mansfield et al., 2021; Treigyte et al., 2024), and mean arterial pressure (MAP) in four studies (Bellini et al., 2025; Cheng et al., 2021; Engelland et al., 2020; Miwa et al., 1994). The effects of repeated hot‐water immersion exposure on resting SBP, DBP, and MAP were analyzed in four studies (Brunt, Howard, et al., 2016; Campbell et al., 2022; Cheng et al., 2025; Cui et al., 2022). Nine studies measured blood pressure using an automated monitor (Bellini et al., 2025; Cheng et al., 2025; Cui et al., 2022; Engelland et al., 2020; Hu et al., 2012; Maley et al., 2023; Miwa et al., 1994; Su et al., 2024; Treigyte et al., 2024), one study employed a manual sphygmomanometer (Campbell et al., 2022), and in two studies, the method was unclear (Brunt, Howard, et al., 2016; Cheng et al., 2021). Blood pressure was measured after seated rest in four studies (Hu et al., 2012; Maley et al., 2023; Mansfield et al., 2021; Treigyte et al., 2024), after supine rest in two studies (Brunt, Howard, et al., 2016; Cui et al., 2022), in the anatomical reference position in one study (Cheng et al., 2025), and two studies did not report the position (Campbell et al., 2022; Cheng et al., 2021). Four studies measured blood pressure in triplicate (Brunt, Howard, et al., 2016; Campbell et al., 2022; Cheng et al., 2021; Hu et al., 2012), one study in duplicate (Mansfield et al., 2021), one study over two or three times (Cui et al., 2022), one study measured in triplicate, but averaged the second and third values (Cheng et al., 2025), one study measured one time (Treigyte et al., 2024), and one study did not specify (Maley et al., 2023).

**FIGURE 7:**
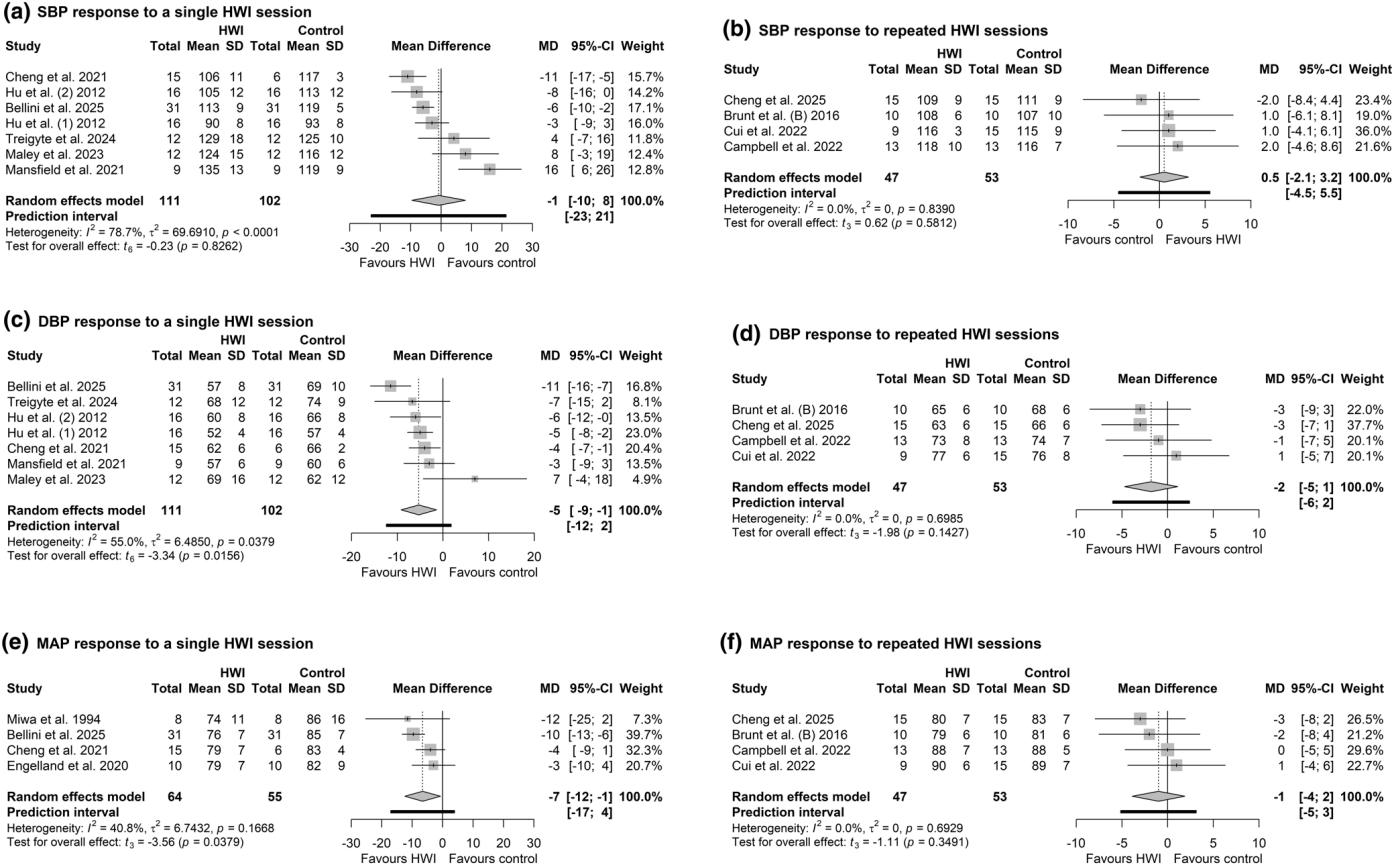
Forest plot of comparison: Hot‐water immersion (HWI) versus control [no HWI or thermoneutral water immersion (TWI)], (a) Systolic blood pressure response at the end of a single HWI session; (b) Systolic blood pressure response after repeated HWI intervention; (c) Diastolic blood pressure response at the end of a single HWI session; (d) Diastolic blood pressure response after repeated HWI intervention (e) Mean arterial pressure response at the end of a single HWI session; (f) Mean arterial pressure response after repeated HWI intervention–random effects analysis.

Single immersion in hot water decreased DBP in 5 [−9, −1] mmHg (PI = −12, 2 mmHg, *p* = 0.01; Figure 7c) and MAP in 7 [−12, −1] mmHg (PI = −17, 4 mmHg, *p* = 0.03; Figure 7e) compared to control conditions. The pooled result for SBP was −1 [−10, 8] mmHg (PI: −23, 21 mmHg, *p* = 0.82; Figure 7a). Additionally, the *I* ^2^ statistics indicated moderate to substantial heterogeneity across the studies for the SBP (*I* ^2^ = 78.7%, *τ* ^2^ = 69.7, *p* < 0.0001; Figure 7a), DBP (*I* ^2^ = 55%, *τ* ^2^ = 6.48, *p* = 0.03; Figure 7c), and MAP (*I* ^2^ = 40.8%, *τ* ^2^ = 6.74, *p* = 0.16; Figure 7e).

For the repeated exposure, the pooled MD for SBP was 0.5 [−2.1, 3.2] mmHg (PI: −4.5, 5.5 mmHg, *τ* ^2^ = 0, *p* = 0.58; Figure 7b), for DBP was −2 [−5, 1] mmHg (PI: −6, 2 mmHg, *τ* ^2^ = 0, *p* = 0.14; Figure 7d), and for MAP was −1 [−4, 2] mmHg (PI: −5, 3 mmHg, *τ* ^2^ = 0, *p* = 0.35; Figure 7f).

A leave‐one‐out analysis across the blood pressure variables showed that the mean difference, effect sizes, and prediction intervals remained consistent across all iterations (Table S1). For the single exposure on SBP, the exclusion of Mansfield et al. (2021) resulted in a pooled effect size of −4.3 [−4.3, −10.9] mmHg (PI = −14.8, 6.2), which reduced, to some extent, the heterogeneity in the findings (*I* ^2^ = 61%, *τ* ^2^ = 12.4).

### 3.6 Stroke volume

Stroke volume was reported in only two studies. Cheng et al. (2021) analyzed stroke volume 45 min after hot‐water immersion, and Cui et al. (2022) after 4 weeks of heat therapy. Cheng et al. (2021) reported that stroke volume was greater in the ankle immersion group compared to the knee immersion, and no change was seen in the control group. However, the data from the intervention and control groups were analyzed separately. Cui et al. (2022) showed no changes in stroke volume after the heating intervention, but no data were reported in the control group.

### 3.7 Plasma volume

One study reported plasma volume data after one and five consecutive hot‐water immersion sessions (Campbell et al., 2022). The change in plasma volume was calculated using the change in hematocrit (Strauss et al., 1951).

The authors demonstrated that water immersion acutely increased plasma volume (%ΔPV) in both hot and thermoneutral water conditions. However, this expansion was blunted after five exposures, regardless of the water temperature.

### 3.8 Cardiac output

Three studies reported cardiac output. Two studies analyzed cardiac output after a single immersion; the method used was unclear (Cheng et al., 2021; Engelland et al., 2020). One study measured cardiac output after four  weeks using transthoracic echocardiography/Doppler (Cui et al., 2022).

**FIGURE 8:**
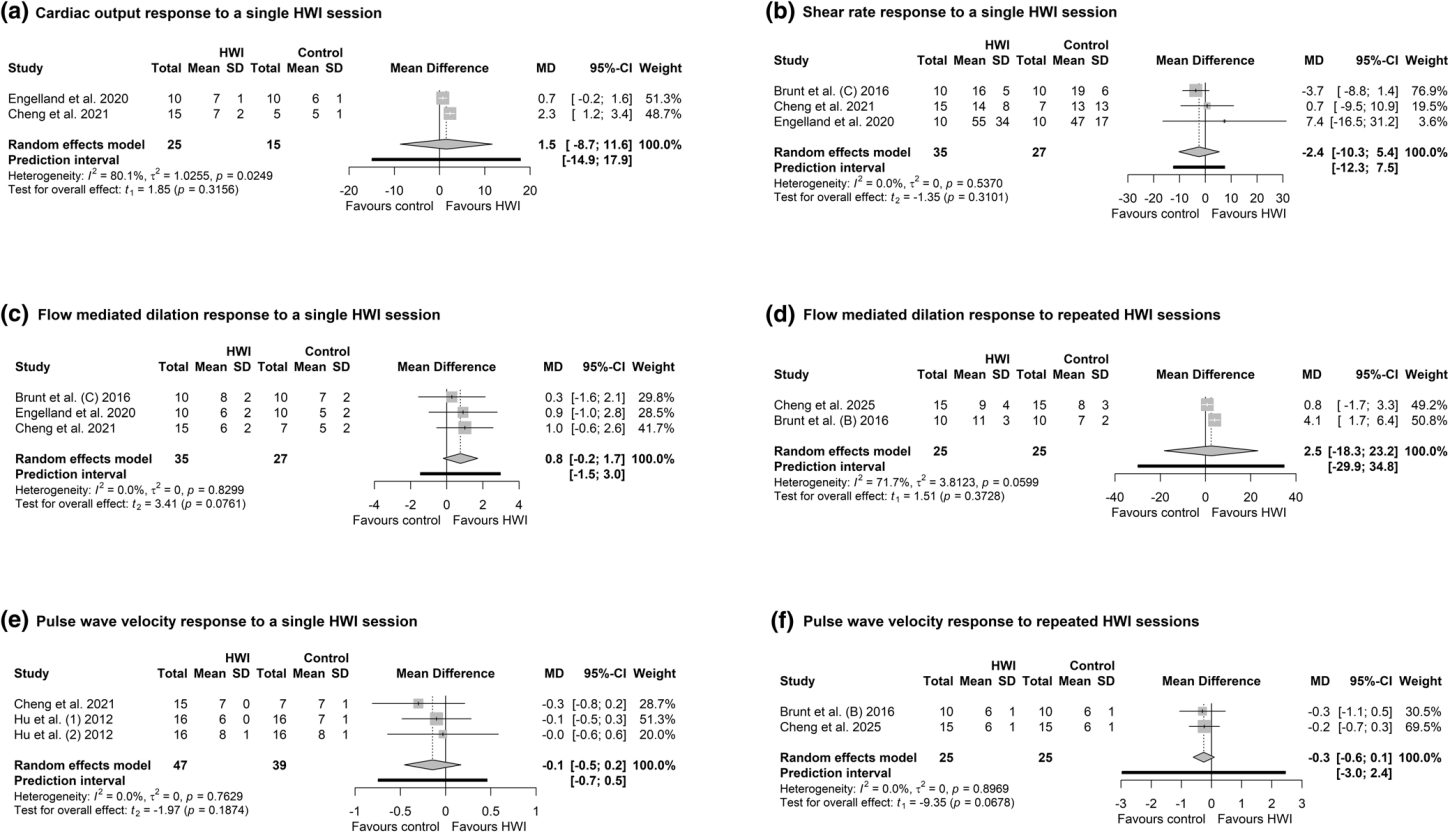
Forest plot of comparison: Hot‐water immersion (HWI) versus control [no HWI or thermoneutral water immersion (TWI)], (a) Cardiac output response at the end of a single HWI session; (b) Shear rate response at the end of a single HWI session; (c) Flow mediated dilation at the end of a single HWI session; (d) Flow mediated dilation after repeated HWI intervention; (e) Pulse wave velocity response at the end of a single HWI session; (f) Pulse wave velocity response after repeated HWI intervention–random effects analysis.

The pooled MD for the cardiac output during short‐term immersion in hot water was 1.5 [−8.7, 11.6] units (PI: −14.9, 17.9 units, *p* = 0.31; Figure 8a), and high heterogeneity was observed (*I* ^2^ = 80.1%, *τ* ^2^ = 1, *p* = 0.02; Figure 8a).

### 3.9 Shear rate

In three studies, the shear rate in the brachial artery was assessed at the end of one immersion session (Brunt, Jeckell, et al., 2016; Cheng et al., 2021; Engelland et al., 2020) and after a repeated intervention in one study (Brunt, Howard, et al., 2016). In all studies, the shear rate was expressed as the area under the shear rate curve.

The pooled MD for the shear rate was −2.4 [−10.3, 5.4] units (PI: −12.3, 7.5, *p* = 0.5; Figure 8c). No heterogeneity was observed (*I* ^2^ = 0%, *τ* ^2^ = 0, *p* = 0.53; Figure 8b). Brunt, Howard, et al. (2016) reported no significant effect on the shear rate between the intervention and control groups. A leave‐one‐out analysis showed that the mean difference, effect sizes, and prediction intervals remained consistent across all iterations (Table S1).

### 3.10 Flow‐mediated dilation

Brachial artery flow‐mediated dilation was assessed at the end of one immersion session in three studies (Brunt, Jeckell, et al., 2016; Cheng et al., 2021; Engelland et al., 2020) and after a repeated intervention in two studies (Brunt, Howard, et al., 2016; Cheng et al., 2025). Reactive hyperemia was induced by inflating a forearm cuff to 200 mmHg (Cheng et al., 2021, 2025), 220 mmHg (Engelland et al., 2020), and 250 mmHg (Brunt, Howard, et al., 2016; Brunt, Jeckell, et al., 2016) for 5 min, followed by deflation. In all studies, flow‐mediated dilation is expressed as the percentage of change in the peak brachial artery diameter relative to the baseline artery diameter.

The pooled MD for flow‐mediated dilation in the single interventions was 0.8 [−0.2, 1.7] units (PI: −1.5, 3 units, *p* = 0.07; Figure 8c). No heterogeneity was observed (*I* ^2^ = 0%, *τ* ^2^ = 0, *p* = 0.82; Figure 8c). For the repeated exposure, the pooled MD was 2.5 [−18.3, 23.2] units (PI: −29.9, 34.8 units, *p* = 0.37; Figure 8d), and substantial heterogeneity was observed (*I* ^2^ = 71.7%, *τ* ^2^ = 3.8, *p* = 0.06; Figure 8d). A leave‐one‐out analysis showed that the mean difference, effect sizes, and prediction intervals remained consistent across all iterations (Table S1).

### 3.11 Arterial stiffness

Pulse wave velocity data were reported after two single (Cheng et al., 2021; Hu et al., 2012) and two repeated exposures (Brunt, Howard, et al., 2016; Cheng et al., 2025). Pulse wave velocity was measured by the carotid‐femoral pulse using tonometry and ultrasound (Brunt, Howard, et al., 2016), simultaneous applanation tonometry (Cheng et al., 2021, 2025), and by the cardio‐ankle vascular index using a vascular screening system (Hu et al., 2012).

The pooled MD for pulse wave velocity in the single intervention was −0.1 [−0.5, 0.2] units (PI: −0.7, 0.5 units, *p* = 0.18; Figure 8e). For the repeated sessions, the pooled effect was −0.3 [−0.6, 0.1] units (PI: −3, 2.4 units, *p* = 0.06; Figure 8f). No heterogeneity was observed in both conditions (*I* ^2^ = 0%, *τ* ^2^ = 0, Figure 8e,f). A leave‐one‐out analysis showed that the mean difference, effect sizes, and prediction intervals remained consistent across all iterations (Table S1).

### 3.12 Cardiorespiratory fitness

Cardiorespiratory fitness was reported in only one study. Cheng et al. (2025) analyzed peak oxygen consumption (VO_2peak_) after 8 weeks of footbaths. The authors reported a mean change of 2.18 mL.kg^−1^.min^−1^ (95% CI: 1.06–4.12) after the heat therapy, but the change scores across the 8‐week intervention between heat therapy and control were not different.

Figure S1 shows the contour‐enhanced funnel plots for all outcomes. The visual inspection for the heart rate response to a single HWI (Figure S1 Panel A) suggests funnel asymmetry, with several studies falling outside the expected funnel shape, indicating a potential small‐study effect. This was confirmed by Egger's regression test (Intercept: 95% CI: 1.06, 10.86, *t* = 2.4, *p* = 0.04; Table S2). Although the studies in all the other outcomes fall within the expected funnel, it is difficult to inspect funnel asymmetry due to the low number of studies. Egger's regression test does not indicate the presence of funnel plot asymmetry. However, it is important to highlight that it is not indicated to perform this test when the number of studies is <10, as it may lack the statistical power to detect bias (Sterne et al., 2011). As such, these findings should be interpreted with caution.

## 4 DISCUSSION

This systematic review examined the effects of single and repeated hot‐water immersion exposures on markers of cardiovascular health and cardiorespiratory fitness. The findings indicated that a single session of hot‐water immersion increases heart rate (when not accounting for between‐study heterogeneity) and reduces diastolic and mean arterial blood pressure, while repeated hot‐water immersion leads to a decrease in resting heart rate. However, due to the limited number of studies included within each variable of interest and the subsequent wide prediction intervals spanning both positive and negative effects, caution is warranted when considering what effects could be expected in the future. Interestingly, only one study reported VO_2_max as an outcome. This scarcity is noteworthy, particularly given recent evidence suggesting that heat therapy may share certain physiological pathways with exercise and could serve as a potential alternative. However, it is important to highlight that many studies did measure key determinants of VO_2_max, such as heart rate, cardiac output, stroke volume, and vascular adaptations.

### 4.1 Quality of studies

Regarding the risk of bias, most randomized studies were judged to have unclear or moderate risk, mostly due to the lack of clarity in the analysis plans (item 5.1 in Domain 5). Only two studies were identified as having a high risk of bias (Kudo et al., 2019; Miwa et al., 1994). Kudo et al. (2019) were classified as having a high risk of bias due to the possible carryover effect (only 10 min were employed between the intervention and control sessions). Miwa et al. (1994) were assessed as having some concerns in three domains, specifically regarding the randomization process, period and carryover effects, and the selection of the reported result. Of the four non‐randomized studies, only two were judged as having a low risk of bias (Brunt, Eymann, et al., 2016; Brunt, Howard, et al., 2016). One (Brazaitis & Skurvydas, 2010) was classified with some concerns due to a lack of information regarding missing data, and one (Cui et al., 2022) was judged as serious, as their data analysis method differed between the intervention and control groups. Although specific tools exist to evaluate the risk of bias in non‐randomized studies, and were used in this study, the value of randomized studies should not be overlooked. Randomization avoids selection bias (Berger et al., 2021) and ensures similarity between groups, which is crucial for making statistical inferences about treatment effects (Lim & In, 2019).

### 4.2 Overall studies characteristics

Twenty laboratory‐based studies were included in the analysis, reporting on 384 participants (210 males,151 females, 23 not specified). Most participants (*n* = 338) were healthy young adults (between 20 and 27 years old), while only 46 were healthy older adults (57–69 years old). This is noteworthy, given the substantial research advocating for the use of passive heat therapies to improve cardiovascular health in various populations (Brunt, Howard, et al., 2016; Kjertakov & Petersen, 2022); most studies have focused on young and healthy subjects. This limitation in the heat therapy research field, particularly in the hot‐water immersion approach, may reflect the relative infancy of this approach. This limited inclusion may also reflect safety considerations, as heat exposure in older or clinical populations can increase the risk of adverse events (Kenny et al., 2010). However, carefully designed protocols could help mitigate these risks and enable future research to assess the therapeutic potential of heat therapy in these populations. Additionally, the underrepresentation of women in thermoregulation (Hutchins et al., 2021) and cardiovascular research (Nathani et al., 2024) is concerning. Given that CVD is the leading cause of mortality for women (Vogel et al., 2021), it is pleasing that the literature evaluating the efficacy of heat therapy in cardiovascular health shows near parity (45% female). This is particularly important, given that biological sex may modulate the cardiovascular and hemodynamic effects of heat exposure (Chaseling et al., 2023; Larson et al., 2021), highlighting the need to accurately report participant characteristics, despite sex‐specific responses not being the focus of this review.

The trials included in this review showed considerable methodological variation, including differences in study design (16 randomized trials and four non‐randomized trials), levels of water immersion (whole‐body or part‐body), duration (10–120 min), total number of sessions (one to 36 sessions), control conditions (thermoneutral water immersion or time control), and water temperatures (37.5°C–45°C for hot‐water immersion, and 33°C–36.5°C for thermoneutral immersion). These variations in protocol designs are critical to consider, as factors such as the duration and temperature, along with the extent of body surface exposure to the heat, can influence the magnitude of physiological adaptations (Crandall & Wilson, 2015). For example, longer immersion times and higher temperatures could be expected to impose greater heat strain and may elicit stronger cardiovascular adaptations. Although it is generally suggested that the core temperature should reach 38.5°C to promote thermoregulatory adaptations (Taylor, 2014), the existence of a threshold for cardiovascular adaptations remains unknown (Brunt & Minson, 2021). Although core temperature more directly indicates thermal stimulus than water temperature or immersion duration, few studies reported its changes, often using varied methods and endpoints, which prevented a formal sub‐analysis (Table S3). We suggest that future research consistently report core temperature with standardized methods to enhance interpretability and reduce heterogeneity.

### 4.3 Intervention effects

The potential health benefits of passive heat therapy, particularly in terms of cardiovascular health, are widely proclaimed (Cheng & MacDonald, 2019; Cullen et al., 2020; Pizzey et al., 2021). However, given the varied methodologies discussed previously, the findings are not unanimous. One of the physiological hallmarks in response to heat stress is cutaneous vasodilation, which redirects blood flow from non‐cutaneous tissues to the skin to dissipate heat (Cheng & MacDonald, 2019). This is facilitated by an increased cardiac output, which is primarily mediated by an increase in heart rate (Cheng & MacDonald, 2019), as stroke volume remains unchanged (Cheng et al., 2021; Cui et al., 2022). Our meta‐analysis showed increased heart rate following a single hot‐water immersion session compared to a control condition. However, given the high heterogeneity (*I* ^2^ = 89.6%, *τ* ^2^ = 143), the results should be interpreted cautiously. The effects of a single hot‐water immersion on blood pressure are inconclusive. Some studies have reported a decrease in mean arterial blood pressure during immersion in hot water (Engelland et al., 2020; Miwa et al., 1994), but this finding is not always observed (Cheng et al., 2021; Kissling et al., 2022). Although the only statistically significant difference found in this meta‐analysis was for DBP and MAP, the true effects remain inconclusive due to the wide prediction intervals observed.

The literature exploring vascular function and structure shows contrasting results (Cheng & MacDonald, 2019). This meta‐analysis provided inconclusive evidence regarding the effects of repeated hot‐water immersion on vascular adaptations such as endothelial function, and arterial stiffness, as reflected by the wide prediction intervals. Shear stress, a mechanistic stimulus induced by immersion, also showed similar ambiguity in its reported effects. Apart from the heterogeneity in the trials, a possible reason for this is the small number of studies included in these analyses (only two or three studies were included for each variable). Thus, the expanded width best reflects the uncertainty of the effect and not necessarily a wide variation in the effect (Borenstein, 2023). Therefore, care should be taken when interpreting these results, and more research is needed to further explore the true effect of hot‐water immersion on vascular outcomes.

The next step in this review was to explore the effects of repeated hot‐water immersion on markers of cardiovascular function and fitness. The meta‐analysis revealed that resting heart rate was reduced after a long‐term intervention compared to a control condition (when heterogeneity was not accounted for). While resting heart rate is known to be influenced by numerous factors and can exhibit considerable variability (Palatini & Julius, 1997), the consistent direction of effect across studies suggests a meaningful physiological trend. Importantly, all measurements were taken under thermoneutral conditions, minimizing the influence of acute heat stress and supporting the interpretation that these reflect chronic adaptations. Repeated exposure to heat stress elicits physiological adaptations that enhance thermoregulation and reduce cardiovascular strain, which can be observed, among other responses, as a lower resting heart rate. These adaptations likely result from a combination of mechanisms, including plasma volume expansion (Tyler et al., 2024), improved autonomic balance (e.g., increased parasympathetic activity) (Ferreira et al., 2023), enhanced vascular function, and reduced systemic resistance (Brunt & Minson, 2021), all of which could contribute to a lower resting heart rate. While previous work (Periard et al., 2015) has highlighted mechanisms such as enhanced skin cooling and blood flow redistribution, we acknowledge that the small number of studies and heterogeneity in heating modalities and participant characteristics introduce uncertainty. Accordingly, we interpret these findings with caution and emphasize the need for further research using standardized protocols.

Regarding the long‐term effects on blood pressure, no effects were observed in systolic, diastolic, or mean arterial pressure. Contrasting results have been found in a previous meta‐analysis (Pizzey et al., 2021), where, compared to a control condition, heat therapy reduced systolic, diastolic and mean arterial pressure, but did not change resting heart rate. However, this discrepancy may stem from methodological differences, as the prior meta‐analysis included various heat stress modalities beyond hot‐water immersion and reported changes from pre‐ to post‐intervention. In contrast, our analysis focused solely on post‐intervention data. Additionally, the small number of studies contributed to wide prediction intervals that encompassed both potential benefits and detriments, underscoring the uncertainty of the results. Considering that a recent meta‐analysis reported reductions of 5 mmHg in systolic blood pressure and 3 mmHg in diastolic blood pressure following aerobic exercise interventions (Edwards et al., 2023), our prediction intervals fall within the clinically relevant range observed in training studies. This is particularly important considering that a 5 mmHg reduction in systolic blood pressure lowers the risk of major cardiovascular events by about 10%, irrespective of baseline cardiovascular status or blood pressure level (Rahimi et al., 2021). Similarly, a 2 mmHg decrease in diastolic blood pressure has been linked to a 6% lower risk of coronary heart disease and a 15% lower risk of stroke (Cook et al., 1995). Our results suggest that hot‐water immersion may potentially lower blood pressure, though further research is needed to confirm this effect.

An important consideration in interpreting the effects of hot‐water immersion is the role of hydrostatic pressure. Unlike air‐based heat therapies, water immersion imposes additional physiological effects through hydrostatic pressure, which can influence venous return, central blood volume, and cardiovascular function. Although relatively few studies have directly compared the physiological responses to water‐ versus air‐based heat therapy, the findings to date are mixed. For example, Campbell et al. (2022) reported that hot water induced less cardiac strain compared to sauna exposure. In contrast, Atencio et al. (2025) reported that HWI elicits greater thermoregulatory and cardiovascular responses compared to traditional and far infrared saunas, as evidenced by a more pronounced increase in core temperature, heart rate, cardiac output, and stroke volume. These contrasting results highlight the complex interplay between thermal load and modality‐specific factors (e.g., hydrostatic pressure and heat transfer efficiency), underscoring the need for a more comprehensive understanding of the physiological effects of different passive heating modalities.

### 4.4 Limitations and future directions

This review is not without limitations. While we tried to separate hot‐water immersion from other modalities of passive heating, we acknowledge the valuable contribution of previous systematic reviews and meta‐analyses that have investigated the cardiovascular benefits of heat therapy across both healthy (Pizzey et al., 2021) and clinical populations (Harwood et al., 2021; Rodrigues, O'Connor, et al., 2025). Our review focused on healthy individuals and a control group as a comparator. This resulted in a limited number of studies eligible for inclusion, which may have underestimated the true effects of this therapy. As such, including clinical populations and pre‐post intervention effects when no control condition is used could provide a more comprehensive understanding of cardiovascular responses to HWI.

The included studies exhibited considerable methodological variation, which likely contributed to the heterogeneity observed in the outcomes. Many of the variables analyzed were secondary or exploratory, potentially leaving some studies underpowered to detect meaningful changes and increasing the risk of type II errors. Additionally, our meta‐analysis relied on post‐intervention data rather than change‐from‐baseline scores due to inconsistent reporting across studies. While this approach enabled broader inclusion, it may have introduced bias from baseline imbalances and reduced precision by ignoring within‐subject changes. These limitations are particularly impactful given the small number of studies included. Further, plasma volume estimates were derived from hematocrit‐based methods, which are indirect, sensitive to hydration status, and less accurate than direct techniques such as carbon monoxide rebreathing. To improve the reliability of future findings, studies should prioritize high‐quality measurement techniques, report both baseline and post‐intervention data, and ensure adequate sample sizes to support robust statistical analysis.

We acknowledge that the advancement of knowledge presented by this review may be considered incremental, given the limited number of eligible studies and variation in protocols. However, by focusing exclusively on HWI, this work helps disentangle modality‐specific effects and offers a more targeted understanding of the cardiovascular and potential fitness‐related responses to repeated heat exposure. Future research should evaluate the optimal parameters, such as duration, frequency, intensity, and extent of body coverage required to elicit meaningful cardiovascular responses to HWI, especially when translating its application to the real world. A frequent limitation in this field is the lack of consistent reporting on adverse events and tolerance issues associated with exposure to high water temperatures (Table 2). Of the 20 studies included in this review, only three reported adverse events, which were generally mild (e.g., transient light‐headedness). Future research should systematically monitor and report adverse events to establish the safety and feasibility of HWI interventions, particularly when used outside supervised settings.

**TABLE 2:** Summary of reported adverse events associated with hot‐water immersion in the studies included in the systematic review and meta‐analysis.

Reported adverse events in HWI	
Study	Adverse events
Baranauskiene et al. (2023)	Not reported.
Bellini et al. (2025)	Not reported.
Brazaitis and Skurvydas (2010)	Not reported.
Brunt, Eymann, et al. (2016)	Subjects tolerated heat therapy well. There were a few reports of lightheadedness, but these typically only occurred within the first one to five sessions.
Brunt, Howard, et al. (2016)	There were some reports of light‐headedness during the initial 20–30 min of full immersion, but these only occurred during the first 1–3 days of exposure.
Brunt, Jeckell, et al. (2016)	Not reported.
Campbell et al. (2022)	Not reported.
Cheng et al. (2021)	Not reported.
Cheng et al. (2025)	Not reported.
Cui et al. (2022)	Not reported.
Eimantas et al. (2022)	Not reported.
Engelland et al. (2020)	Not reported.
Hu et al. (2012)	Not reported.
Kingma et al. (2021)	Three participants completed the study but experienced orthostatic hypotension while exiting the bath.
Kudo et al. (2019)	Not reported.
Maley et al. (2023)	Not reported.
Mansfield et al. (2021)	Not reported.
Miwa et al. (1994)	Not reported.
Su et al. (2024)	Not reported.
Treigyte et al. (2024)	Not reported.

It was somewhat surprising that only one study reported data on cardiorespiratory fitness, considering the increasing interest in the exercise‐like effects of heat therapy and its potential as an alternative or adjunct to exercise, especially for vulnerable populations (e.g., clinical groups, older adults, or individuals with low exercise tolerance). Again, this lack of data is partially attributable to our inclusion criteria, which excluded two studies that assessed cardiorespiratory fitness after repeated HWI, even though this was not the primary outcome of either paper. Importantly, this review highlights critical gaps in the literature, most notably the scarcity of studies assessing cardiorespiratory fitness outcomes (e.g., VO_2_max) using appropriate control groups. Addressing these gaps through well‐controlled, longitudinal studies will be essential for determining the potential role of HWI as a therapeutic or performance‐enhancing intervention. Cardiorespiratory fitness is a marker of total body health and is associated with health outcomes, with recent studies suggesting a protective effect of cardiorespiratory fitness over cardiovascular disease risks and mortality (Al‐Mallah et al., 2018). For example, cohort studies report an improvement in survival rates and a decreased incidence of cardiovascular disease with higher cardiorespiratory fitness levels (Al‐Mallah et al., 2014; Berry et al., 2011; Cremer et al., 2017; Kokkinos et al., 2010; Laukkanen et al., 2001; Lee et al., 2011), demonstrating its critical role in preventing cardiovascular morbidity and mortality. Interestingly, cardiorespiratory fitness seems to be a stronger predictor of mortality risk than traditional risk factors assessed clinically, such as blood pressure, heart rate, body mass index, and blood profile (Ross et al., 2016). Given the relevance of this variable, future research should investigate whether and to what extent repeated HWI sessions can improve cardiorespiratory fitness.

## 5 CONCLUSION

This systematic review and meta‐analysis demonstrated that a single hot‐water immersion session may increase heart rate and decrease diastolic and mean arterial blood pressure, while repeated exposure appears to reduce resting heart rate. However, due to the limited number of studies, methodological heterogeneity, and wide prediction intervals, these findings remain inconclusive. This uncertainty is particularly relevant given recent interest in heat therapy as a potential exercise mimetic, especially for populations with limited exercise capacity. The scarcity of data on cardiorespiratory fitness is notable and highlights a critical gap in the literature. Future research should prioritize standardized protocols incorporating the FITT principles (Frequency, Intensity, Time, and Type). This includes clearly describing how often sessions are conducted, the temperature and physiological response achieved, the duration of each session, and the method and body coverage of immersion (for review, see Rodrigues, Minett, & Orssatto (2025)). Such standardization would allow for clearer interpretation and more meaningful comparison across studies. In addition, future work should include more diverse and clinically relevant populations, and explore the long‐term effects of hot‐water immersion on both cardiovascular and cardiorespiratory outcomes. Such efforts are crucial for clarifying the therapeutic potential of HWI and informing its application in health promotion and disease prevention.

## AUTHOR CONTRIBUTIONS

Concept and design: Bruna Bittencourt Sotomaior, Ian B. Stewart, Raul Osiecki, and Geoffrey M. Minett. Acquisition, analysis or interpretation of data: Bruna Bittencourt Sotomaior, Ian B. Stewart, Patrick Rodrigues, and Geoffrey M. Minett. All authors contributed to manuscript drafting and revision. All authors have approved the final version of the manuscript and agree to be accountable for all aspects of the work in ensuring that questions related to the accuracy or integrity of any part of the work are appropriately investigated and resolved. All persons designated as authors qualify for authorship, and all those who qualify for authorship are listed.

## FUNDING INFORMATION

No funding was received for the preparation of this article.

## CONFLICT OF INTEREST STATEMENT

The authors report there are no competing interests to declare.

## Supporting information


**Table S1.** Leave‐one‐out analysis for all variables where the pooled effects and estimates of heterogeneity were recalculated with one study omitted each time.

**Table S2.** Egger’s linear regression test for funnel plot asymmetry.

**Table S3.** Difference between pre‐ and post‐immersion core temperature for single exposure studies, and difference in resting core temperature (e.g., baseline vs. final session) for repeated exposure studies.

**Data S1.** Prisma 2020 Checklist.

**Data S2.** Complete list of search terms.

**Figure S1.** Counter‐enhanced funnel plots to inspect funnel plot asymmetry.

## Data Availability

Data will be made available upon reasonable request.
