# Supplementary material for: The effects of hot‐water immersion on cardiovascular and cardiorespiratory health of healthy adults: A systematic review and meta‐analysis
Source: Physiol Rep. 2026 Jan 28;14(2):e70668. doi: 10.14814/phy2.70668 (PMC12848596; doi:10.14814/phy2.70668)
Supplement: Supplementary file 1 — Table S1. Leave‐one‐out analysis for all variables where the pooled effects and estimates of heterogeneity were recalculated with one study omitted each time. [file PHY2-14-e70668-s006.docx]

Table S1: Leave-one-out analysis for all variables where the pooled effects and estimates of heterogeneity were recalculated with one study omitted each time.

| **Variable** | **Study left out** | **MD** | **Lower 95%CI** | **Upper 95%CI** | **I-squared** | **Tau-squared** | **Lower PI** | **Upper PI** |
| --- | --- | --- | --- | --- | --- | --- | --- | --- |
| HR – single HWI session | Maley et al. | 26 | 17 | 36 | 0.89 | 127 | -1 | 53 |
|  | Miwa et al. | 26 | 17 | 36 | 0.89 | 128 | -1 | 53 |
|  | Treigyte et al. | 26 | 17 | 34 | 0.88 | 103 | 1 | 50 |
|  | Mansfield et al. | 26 | 17 | 35 | 0.90 | 118 | 0 | 52 |
|  | Cheng et al. | 27 | 18 | 37 | 0.90 | 139 | -1 | 55 |
|  | Su et al. | 27 | 18 | 37 | 0.90 | 143 | -1 | 56 |
|  | Kingma et al. | 28 | 19 | 38 | 0.91 | 141 | 0 | 57 |
|  | Bellini et al.  Engelland et al.  Hu et al. (1)  Hu et al. (2) | 28  29  29  29 | 19  20  20  21 | 38  38  38  38 | 0.90  0.90  0.90  0.86 | 139  127  127  101 | 0  2  2  5 | 57  56  56 |
|  |  |  |  |  |  |  |  | 54 |
| HR – repeated HWI sessions | Brunt et al. (A) | -4 | -6 | -1 | 0 | 0 | -9 | 1 |
|  | Brunt et al. (B) | -4 | -7 | -1 | 0 | 0 | -9 | 2 |
|  | Cui et al. | -4 | -7 | -1 | 0 | 0 | -10 | 2 |
|  | Campbell et al.  Cheng et al. | -3  -3 | -6  -5 | 0  -1 | 0  0 | 0  0 | -8  -8 | 2  2 |
| SBP – single HWI session | Mansfield et al. | -4.3 | -10.9 | 2.3 | 0.61 | 12.4 | -14.8 | 6.2 |
|  | Maley et al. | -2.3 | -12.2 | 7.6 | 0.78 | 52.0 | -22.7 | 18.2 |
|  | Treigyte et al. | -1.6 | -12.1 | 8.9 | 0.81 | 61.6 | -23.7 | 20.6 |
|  | Hu et al. (1) | -0.4 | -11.4 | 10.6 | 0.82 | 70.9 | -24.2 | 23.4 |
|  | Bellini et al.  Hu et al. (2)  Cheng et al. | 0.2  0.3 | -10.5  -10 | 10.9  10.6 | 0.81  0.82 | 66.4  61.4 | -22.9  -21.9 | 23.3  22.5 |
|  |  | 0.8 | -8.7 | 10.3 | 0.78 | 47.6 | -18.9 | 20.5 |
| SBP – repeated HWI sessions | Campbell et al. | 0.1 | -4.1 | 4.3 | 0.00 | 0.0 | -7.5 | 7.7 |
|  | Cui et al. | 0.2 | -5.5 | 5.6 | 0.00 | 0.0 | -8.2 | 8.7 |
|  | Brunt et al. (B) | 0.4 | -4.4 | 5.2 | 0.00 | 0.0 | -7.1 | 7.9 |
|  | Cheng et al. | 1.3 | -0.1 | 2.6 | 0.00 | 0.0 | -6.4 | 9 |
| DBP – single HWI session | Maley et al. | -5.8 | -9.0 | -2.7 | 0.42 | 2.4 | -10.8 | -0.9 |
|  | Cheng et al. | -5.7 | -10.5 | -0.8 | 0.60 | 5.8 | -13 | 1.7 |
|  | Mansfield et al. | -5.7 | -10.2 | -1.2 | 0.60 | 4.3 | -12.1 | 0.7 |
|  | Hu et al. (1) | -5.3 | -10.5 | -0.2 | 0.62 | 7.6 | -13.6 | -2.9 |
|  | Hu et al. (2)  Treigyte et al.  Bellini et al. | -5.2  -5.2 | -9.9  -9.7 | -0.5  -0.8 | 0.62  0.62 | 5.8  4.7 | -12.4  -11.8 | -2.0  1.3 |
|  |  | -4.4 | -6.8 | -2.0 | 0.00 | 0.0 | -6.8 | -2.0 |
| DBP – repeated HWI sessions | Cui et al. | -2.5 | -5.1 | 0.1 | 0.00 | 0.0 | -8.9 | 3.9 |
|  | Campbell et al. | -2.0 | -7.2 | 3.2 | 0.00 | 0.0 | -8.4 | 4.4 |
|  | Brunt et al. (B) | -1.5 | -6.5 | 3.5 | 0.00 | 0.0 | -7.9 | 5 |
|  | Cheng et al. | -1.1 | -6.0 | 3.8 | 0.00 | 0.0 | -8.3 | 6.1 |
| MAP – single HWI session | Cheng et al. | -8.4 | -16.7 | 0 | 0.30 | 0.0 | -15.4 | -1.4 |
|  | Engelland et al. | -7.5 | -16.3 | 1.2 | 0.45 | 2.4 | -17.8 | 2.7 |
|  | Miwa et al.  Bellini et al. | -6.4  -4.3 | -15.5  -10.7 | 2.8  2.2 | 0.57  0.00 | 3.7  0.0 | -17.7  -12.6 | 5.0  4.1 |
| MAP – repeated HWI sessions | Cui et al. | -1.6 | -5.5 | 2.4 | 0.00 | 0.0 | -7.9 | 4.8 |
|  | Campbell et al. | -1.4 | -6.6 | 3.7 | 0.00 | 0.0 | -8.1 | 5.2 |
|  | Brunt et al. (B) | -0.3 | -3.8 | 3.3 | 0.00 | 0.0 | -6.8 | 6.2 |
| CO – single HWI session | Cheng et al.  Engelland et al. | 0.7  2.3 | -0.2  1.2 | 1.6  3.4 | NA  NA | NA  NA | NA  NA | NA  NA |
| FMD – single HWI session | Cheng et al. | 0.6 | -.34 | 4.5 | 0.00 | 0.0 | -8.0 | 9.1 |
|  | Cheng et al. | 0.7 | -3.8 | 5.2 | 0.00 | 0.0 | -7.0 | 8.4 |
|  | Engelland et al. | 1.0 | 0.3 | 1.6 | 0.00 | 0.0 | -6.8 | 8.7 |
| FMD – repeated HWI sessions | Brunt et al. (B)  Cheng et al. | 1  4 | -2  2 | 3  6 | NA  NA | NA  NA | NA  NA | NA  NA |
| SR – single HWI session | Cheng et al. | -3 | -32 | 26 | 0 | 0 | -36 | 29 |
|  | Engelland et al. | -3 | -25 | 20 | 0 | 0 | -33 | 27 |
|  | Brunt et al. (C) | 2 | -29 | 32 | 0 | 0 | -59 | 63 |
| PWV – single HWI session | Hu et al. (1) | -0.2 | -1.9 | 1.9 | 0 | 0 | -2.7 | 2.4 |
|  | Hu et al. (2) | -0.2 | -1.4 | 1.0 | 0 | 0 | -2.2 | 1.8 |
|  | Cheng et al. | -0.1 | -0.5 | 0.3 | 0 | 0 | -2.2 | 2.0 |
| PWV – repeated HWI sessions | Cheng et al. | -0.3 | -1.1 | 0.5 | NA | NA | NA | NA |
|  | Brunt et al. (B) | -0.2 | -0.7 | 0.3 | NA | NA | NA | NA |
