# Supplementary material for: The effects of hot‐water immersion on cardiovascular and cardiorespiratory health of healthy adults: A systematic review and meta‐analysis
Source: Physiol Rep. 2026 Jan 28;14(2):e70668. doi: 10.14814/phy2.70668 (PMC12848596; doi:10.14814/phy2.70668)
Supplement: Supplementary file 2 — Table S2. Egger’s linear regression test for funnel plot asymmetry. [file PHY2-14-e70668-s003.docx]

Table S2: Egger’s linear regression test for funnel plot asymmetry.

| **Outcome** | **Intercept** | **95% CI** | **t-value** | **p-value** |
| --- | --- | --- | --- | --- |
| HR – single HWI session | 5.96 | 1.06, 10.86 | 2.38 | 0.04 |
| HR – repeated HWI sessions | -0.27 | -2.46, 1.92 | -0.24 | 0.82 |
| SBP – single HWI session | 3.16 | -0.19, 6.51 | 1.85 | 0.12 |
| SBP – repeated HWI sessions | -0.22 | -5.3, 4.85 | -0.08 | 0.93 |
| DBP – single HWI session | 0.83 | -2.19, 3.84 | 0.54 | 0.61 |
| DBP – repeated HWI sessions | 2.93 | -1.48, 7.35 | 1.30 | 0.32 |
| MAP – single HWI session | 0.56 | -3.34, 4.46 | 0.28 | 0.80 |
| MAP – repeated HWI sessions | -0.37 | -13.15, 12.4 | -0.06 | 0.95 |
| FMD – single HWI session | -2.32 | -8.86, 4.21 | -0.69 | 0.61 |
| SR – single HWI session | 1.28 | 0.79 – 1.78 | 5.05 | 0.12 |
| PWV – single HWI session | -0.13 | -4.43, 4.16 | -0.06 | 0.96 |
