# Supplementary material for: The effects of hot‐water immersion on cardiovascular and cardiorespiratory health of healthy adults: A systematic review and meta‐analysis
Source: Physiol Rep. 2026 Jan 28;14(2):e70668. doi: 10.14814/phy2.70668 (PMC12848596; doi:10.14814/phy2.70668)
Supplement: Supplementary file 3 — Table S3. Difference between pre‐ and post‐immersion core temperature for single exposure studies, and difference in resting core temperature (e.g., baseline vs. final session) for repeated exposure studies. [file PHY2-14-e70668-s004.docx]

Table S3: Difference between pre- and post-immersion core temperature for single exposure studies, and difference in resting core temperature (e.g., baseline vs final session) for repeated exposure studies.

| Authors (yr) | Groups | ∆ Core temperature (°C) | Method |
| --- | --- | --- | --- |
| Baranauskiene (2023) | HWI | 2.39 | Rectal thermistor |
|  | Control | 2.39 |  |
| Bellini (2024) | HWI | 1 | Rectal thermistor or telemetric pill |
|  | Control | 0 |  |
| Brazaitis (2010) | HWI | -0.3 | Rectal thermistor |
|  | Control | - |  |
| Brunt (2016a) | HWI | -0.4 | Rectal thermistor |
|  | Control | -0.1 |  |
| Brunt (2016b) | HWI | -0.2 | Rectal thermistor |
|  | Control | 0 |  |
| Brunt (2016c) | HWI | 0.4 | Rectal thermistor |
|  | Control | -0.2 |  |
| Campbell (2022) | HWI | -0.2 | Rectal thermistor |
|  | Control | -0.1 |  |
| Cheng (2021) | HWI | 0.4 | Gastrointestinal – Telemetric pill |
|  | HWI | 0.7 |  |
|  | Control | -0.2 |  |
| Cheng (2025) | HWI | -0.01 | Gastrointestinal – Telemetric pill |
|  | Control | 0.07 |  |
| Cui (2022) | HWI | - | - |
|  | Control | - |  |
| Eimantas (2022) | HWI | 0.2 | Rectal thermistor |
|  | Control | 0 |  |
|  | Control | - |  |
| Engelland (2020) | HWI | 0.7 | Gastrointestinal – Telemetric pill |
|  | Control | 0.2 |  |
| Hu (2012) | HWI | Young: 0 / Older: 0 | Tympanic |
|  | Control | Young: 0 / Older: -0.1 |  |
| Kingma (2021) | HWI | 1.2 | Gastrointestinal |
|  | Control | 0.1 |  |
| Kudo (2019) | HWI | - | - |
|  | Control | - |  |
| Maley (2023) | HWI | 1.6 | Rectal thermistor |
|  | Control | 0.1 |  |
| Mansfield (2021) | HWI | 1.4 | Rectal thermistor |
|  | Control | 0.1 |  |
| Miwa (1994) | HWI | - | - |
|  | Control | - |  |
| Su (2024) | HWI | 0.6 | Rectal thermistor |
|  | Control | -0.3 |  |
| Treigyte (2024) | HWI | 1.4 | Rectal thermistor |
|  | Control | 0 |  |

Notes: Brunt 2016(b): average temperature during first and last sessions; Cui: not reported; Kudo (2019): reported an increase of ~0.1; Miwa (1994): data reported but not extractable.
