# Supplementary material for: The effects of hot‐water immersion on cardiovascular and cardiorespiratory health of healthy adults: A systematic review and meta‐analysis
Source: Physiol Rep. 2026 Jan 28;14(2):e70668. doi: 10.14814/phy2.70668 (PMC12848596; doi:10.14814/phy2.70668)
Supplement: Supplementary file 4 — Data S1. Prisma 2020 Checklist. [file PHY2-14-e70668-s002.docx]

**Appendix S2 - Complete List of Search Terms**

**PubMed**

((((((immersion[MeSH Terms]) OR (water[MeSH Terms])) OR (bath[MeSH Terms])) AND ((hot temperature[MeSH Terms]) OR (hyperthermia, induced[MeSH Terms]))) OR (((((("hot water immersion"[Title/Abstract]) OR ("passive heat*"[Title/Abstract])) OR ("heat stress"[Title/Abstract])) OR ("heat therapy"[Title/Abstract])) OR ("heat acclimation"[Title/Abstract])) OR ("heat exposure"[Title/Abstract]))) AND ((((((((((blood pressure[MeSH Terms]) OR (cardiac output[MeSH Terms])) OR (stroke volume[MeSH Terms])) OR (heart rate[MeSH Terms])) OR (plasma volume[MeSH Terms])) OR (blood plasma volume[MeSH Terms])) OR (blood flow velocity[MeSH Terms])) OR (arterial stiffness[MeSH Terms])) OR (cardiorespiratory fitness[MeSH Terms])) OR (((((((((((cardiovascular*[Title/Abstract]) OR ("endothelial function"[Title/Abstract])) OR ("shear rate"[Title/Abstract])) OR ("artery flow mediated dilation"[Title/Abstract])) OR ("artery flow mediated dilatation"[Title/Abstract])) OR ("artery diameter"[Title/Abstract])) OR ("oxygen consumption"[Title/Abstract])) OR ("maximal oxygen consumption"[Title/Abstract])) OR (vo2[Title/Abstract])) OR (vo2max[Title/Abstract])) OR ("aerobic fitness"[Title/Abstract])))) NOT ((animals[MeSH Terms]) NOT (humans[MeSH Terms]))

**Web of Science**

(TS=(“hot water immersion” OR “passive heat*” OR “heat stress” OR “heat therapy” OR “heat acclimation” OR “heat exposure”)) AND TS=(“blood pressure” OR “cardiac output” OR “stroke volume” OR “heart rate” OR “plasma volume” OR “blood flow velocity” OR “shear rate” OR “arterial stifness” OR “endothelial function” OR “artery flow mediated dilatation” OR “artery flow mediated dilation” OR “artery diameter” OR cardiovascular* OR “cardiorespiratory fitness” OR “oxygen consumption”)

**Scopus**

( ( ( TITLE-ABS ( “hot water immersion” OR “passive heat*” OR “heat stress” OR “heat therapy” OR “heat acclimation” OR “heat exposure” ) ) AND ( TITLE-ABS ( “blood pressure” OR “cardiac output” OR “stroke volume” OR “heart rate” OR “plasma volume” OR “blood flow velocity” OR “shear rate” OR “arterial stiffness” OR “endothelial function” OR “artery flow mediated dilatation” OR “artery flow mediated dilation” OR “artery diameter” OR cardiovascular* OR “cardiorespiratory fitness” OR “oxygen consumption” ) ) ) AND ( INDEXTERMS ( human ) ) ) AND NOT ( INDEXTERMS ( animal ) )

**CINAHL**

((TI “hot water immersion” OR “passive heat*” OR “heat stress” OR “heat therapy” OR “heat acclimation” OR “heat exposure” OR AB “hot water immersion”) OR (“passive heat*” OR “heat stress” OR “heat therapy” OR “heat acclimation” OR “heat exposure”)) AND ((TI “blood pressure” OR “cardiac output” OR “heart rate” OR “stroke volume” OR “plasma volume” OR “blood flow velocity” OR “arterial stiffness” OR cardiovascular* OR “endothelial function” OR “shear rate” OR “artery flow mediated dilatation” OR “artery flow mediated dilation” OR “cardiorespiratory fitness” OR “oxygen consumption” OR “maximal oxygen uptake”) OR (AB “blood pressure” OR “cardiac output” OR “heart rate” OR “stroke volume” OR “plasma volume” OR “blood flow velocity” OR “arterial stiffness” OR cardiovascular* OR “endothelial function” OR “shear rate” OR “artery flow mediated dilatation” OR “artery flow mediated dilation” OR “cardiorespiratory fitness” OR “oxygen consumption” OR “maximal oxygen uptake”))

**EMBASE**

(('hot water immersion'/exp OR 'hot water immersion':ab,ti OR 'thermotherapy':ab,ti OR 'passive heating':ab,ti OR 'passive heat*':ab,ti OR 'heat exposure':ab,ti OR 'thermal exposure':ab,ti OR 'heat acclimation':ab,ti) AND ('blood pressure':ab,ti OR 'cardiac output':ab,ti OR 'heart output':ab,ti OR 'heart stroke volume':ab,ti OR 'heart rate':ab,ti OR 'plasma volume':ab,ti OR 'blood flow velocity':ab,ti OR 'arterial stiffness':ab,ti OR cardiovascular*:ab,ti OR 'endothelial function':ab,ti OR 'shear rate':ab,ti OR 'artery flow mediated dilatation':ab,ti OR 'artery flow mediated dilation':ab,ti OR 'cardiorespiratory fitness':ab,ti OR 'oxygen consumption':ab,ti OR 'maximal oxygen uptake':ab,ti))

**SPORTDiscus**

((TI “hot water immersion” OR “passive heat*” OR “heat stress” OR “heat therapy” OR “heat acclimation” OR “heat exposure”) OR (AB “hot water immersion” OR “passive heat*” OR “heat stress” OR “heat therapy” OR “heat acclimation” OR “heat exposure”) AND (TI “blood pressure” OR “cardiac output” OR “heart rate” OR “stroke volume” OR “plasma volume” OR “blood flow velocity” OR “arterial stiffness” OR cardiovascular* OR “endothelial function” OR “shear rate” OR “artery flow mediated dilatation” OR “artery flow mediated dilation” OR “cardiorespiratory fitness” OR “oxygen consumption” OR “maximal oxygen uptake”) OR (AB “blood pressure” OR “cardiac output” OR “heart rate” OR “stroke volume” OR “plasma volume” OR “blood flow velocity” OR “arterial stiffness” OR cardiovascular* OR “endothelial function” OR “shear rate” OR “artery flow mediated dilatation” OR “artery flow mediated dilation” OR “cardiorespiratory fitness” OR “oxygen consumption” OR “maximal oxygen uptake”))
